# Extra Supplement—The Nursing Mirror

**Published:** 1889-09-14

**Authors:** 


### The Nursing Mirror.

All Contributions for this Supplement should be addressed to the Editor, The Hospital, 139-140, Salisbury Court, Fleet Street, London, E.C., and should have the word "Nursing" plainly written in left-hand top corner of the envelope

### En Passant.

LADY DOCTORS.—Do women like to be attended in illness by their own sex? This is a subject on which the *Munich Ladies' Journal* has opened its columns for a plébiscite. It asks, "In the event of the illness either of yourself or your children, would you wish to be treated by a lady doctor, and on what grounds do you found your reply?" The result of this novel poll is to be published, and it is to be hoped that the answers will be very numerous. Men are found who state that women will not trust their own sex in any critical crisis.

DOLL COMPETITION.—In reply to numerous enquirers we have to state (1) that no dolls will be returned at all, at first, or until the exhibition or exhibitions are finally closed; (2) that all competitors whose dolls are permanently retained will have the option of receiving a free copy of The Hospital for twelve months; (3) the latest date on which dolls will be received is the 30th November, 1889; (4) all dolls intended for competition are to be addressed to the Editor, The Lodge, Porchester-square, London, W., carriage paid, marked in left-hand top corner, "Doll Competition." It has been decided to keep the list of competitors open until 31st October next.

REGISTRATION.—Dr. Gerald Harper has written a sensible letter to the *British Medical Journal*, in which he points out once more the dangers of a register of all those who have practised nursing for three years. Even if at the time of registration a nurse is capable, who is to be responsible for her fitness ten years later? In ten years a woman may forget her nursing knowledge, and sink to terrible depths of degradation, yet her name would remain on the register with the names of some of the best and noblest nurses in the land. There is little chance, we are glad to say, of this register ever being carried out, for doctors and nurses alike are being aroused to a sense of the danger it would entail, and are ready to oppose it, if ever practical measures are taken to start it.

MASSAGE.—A constant question amongst nurses just now is "What is the Weir Mitchell treatment? How does it differ from ordinary massage?" Nearly every nurse now has some knowledge of massage. It has come to be included amongst the things demanded of her by the doctors under whom she works; but in many cases the knowledge is very slight, and nearly all theoretical. Weir Mitchell treatment includes, besides massage, isolation, rest, diet, and sometimes galvanism. The Weir Mitchell patient is taken far from her friends and allowed no visitors, no letters, no excitement, no exercise. She generally spends her days dosing in bed, the only variety being, perhaps, massage for twenty minutes night and morning. Some wretched, nervous skeleton, worn to a thread, according to the common phrase, will grow fat and composed under a course of this treatment. Diet, of course, differs in different cases, but usually consists of large quantities of milk, beef juice, and other light food, given at frequent intervals. It largely rests with the nurse to persuade the patient to fully carry out the Weir Mitchell treatment, to take the food, and to abstain from all movement or mental worry. The nurse who attends and has charge of the patient should not also act as masseuse. The Weir Mitchell treatment is specially useful in cases of anæmia, dipsomania, or nervous diseases generally.

SEAMEN'S HOSPITAL.—We extract the following paragraph from the late report of this hospital: "The nursing of the hospital is all that can be desired, and your committee feel that it is conducted with full regard to all the details of modern requirements. Ten probationers have been trained during the year. A separate sitting-room and a recreation ground have been provided for the nurses."

SICK CHILDREN IN HOLLAND.—At the Hague there is a hospital for children, under the direction of Mdlle. A. J. I. van Deinse, assisted by two trained nurses; at Scheveningen, quite near by, is an establishment for poor scrofulous children. Rotterdam has its hospital for children, under the direction of Madame Simon van der Aa, the widow of a lamented marine officer. Attached to it is a school for the training of nurses, practically and theoretically. The Red Cross Society also looks after invalid children, for in Holland, as elsewhere, the cry of a child in pain appeals to every woman's heart.

CHARITY AND RELIGION.—A French bishop has been grievously shocked because some ladies, not being members of a sisterhood, thought they had a right to tend the sick. Two societies of ladies for relieving wounded soldiers were founded some years ago—that of Les Dames de France and that of Les Femmes de France. The former are headed by Maréchale MacMahon, and are aristocratic, while the Femmes de France are wealthy and active Republican ladies, among whom there are not a few Protestants and Jewesses. Their president is Madame Koechlin-Schwartz, who has been prominent for years in the cremation movement, and Mesdames Floquet and Jules Ferry are vice-presidents. Some ladies of the Dames de France at Toulouse wished also to belong to the other organisation, and consulted the Bishop of Carcassonne on the subject. He was nothing less than scandalised, and wrote a letter intended for all the ladies of his diocese to say that the women of France only wanted to show that ladies could be as useful as sisters of charity. The latter had been turned out of the schools and hospitals, and there was now an attempt to turn them out of the ambulances under the beautiful mask of patriotism and philanthropy.

AIR SPACE.—An indignant nurse lately told an examiner, who had asked her what air space she would allow for a patient, that she didn't consider that was a question which had anything to do with nursing. Yet, without plenty of pure air a patient is not likely to recover, and the nurse who is constantly by the bed should be responsible for the quantity and quality of the air her patient breathes. Every nurse while training should take her foot-rule and measure the wards in which she works; then counting the beds, by a proportion sum, she can find out the cubic feet allowed to each bed. To a cottage-hospital matron such knowledge is absolutely essential. Prussian army regulations allow only 1,200 cubic feet to a bed in medical wards. English rules for garrison hospitals give 2,000, and at Massachusetts every patient is provided with 3,000 cubic feet of air. In private nursing, a nurse should remember that though there is only one bed in the room, it may constantly be occupied by three persons: herself, her patient, and the patient's nearest relative, so that air space should be allowed for three people. It is always safe to allow as much air as possible; it is never safe to nurse a patient who has less than 2,000 cubic feet of air to breathe.

### Physiology for Nurses.

### NO. IX.—DISLOCATIONS.

But though the hip-joint is liable to disease, this is not the case with all ball and socket joints ; at the shoulder, for instance, the large bone of the arm is very likely to be "put out of joint," as it is called—that is, these ligaments are so strained by violence that the head of the bone is pulled out of its socket. This is an accident which is very likely to happen if children are lifted up by one arm, and very painful it is, too. The feet, again, are made up of a great many bones, which help to make walking easier for us. If our foot was made of only one bone our walking would be very clumsy. The foot, when it has not been spoiled by badly made shoes, is made in the form of an arch, which, you remember, is the form which gives most strength, and as the feet are to support the body you want strength ; also Nature makes the foot broad, so as to have more surface over which to distribute the weight, only people are so foolish sometimes, and so conceited, that they think they can do better for themselves than Nature has done, and they do not like their feet to be so wide, so they squeeze them up in ill-shapen boots. However, Nature usually revenges herself on those who ill-treat her, and one result, and a very painful one, too, of this squeezing is the appearance of corns. What they are and why they come you will not understand until we come to the skin and its structure ; but there is no denying the fact that they are most painful and troublesome, and when too late the poor victim bitterly repents of the folly which made her indulge in elegant rather than sensible boots. The natural arch of the foot, and, therefore, of course, also part of the strength, is lost by wearing too high heels. Each of these bones in the ankle and the foot itself has its proper place and its own work to do, and is also so formed as to bear the exact amount of the weight of the whole body which is its proper share, but if you raise the heel you put the bones out of their proper position, and throw too much weight on the lower end of the foot, and so the foot will come down flat. A very painful thing it is to be "flat-footed," as it is called. Man is the only creature who can stand firmly on his feet and hold himself quite erect, and also move in that position ; though a species of monkey can nearly do so, but not so perfectly as man can. You know how the Chinese ladies treat their women's feet. When the little girl is a baby they tie up her feet very firmly, so as to prevent them from growing, and therefore are shaped something like a ball, with very short toes indeed ; and when these women grow up they can hardly move about at all on them.

Before we quite take leave of our bones, I should like just to tell you, as shortly as possible, the way in which a bone grows, and the different stages through which it goes from its birth to its death ; and we will choose the thigh-bone and go through its history. In a very young infant —before it is born—this bone is not bone at all, but a mass of cartilage or gristle, but something of the shape which the bone is to be afterwards ; this gristle is of a beautiful pearly-white colour, and looks just like the substance which you see in a fresh joint. In this soft gristle a great many bloodvessels are found, which bring blood to it and nourish it, and the gristle has the power of taking out of the blood just what it wants for its own nourishment and passing over everything else. This gristle is soft and yielding now, and would be a very poor support for the body, and it is necessary to make it hard and into bone, and what must it choose from the blood to do this ? In the blood are mixed certain salts and earthy matters, and especially an earthy matter called phosphate of lime ; and though to you and me the blood looks red, and we should not be able to guess that these hard materials are flowing about in solution, still the gristle or cartilage has the power of attracting them and taking possession of them ; and so they get deposited, as it is called, and you will find places in the cartilage where the phosphate of lime has remained. More and more is attracted by the gristle, and these "ossifying centres," as they are called, spread and spread until they meet one another, and so spread, until all over the gristle there is a hard yellowish-white material, which is bone. Thus nearly all the bones in the body are formed, and you will see that they are composed of the two distinct parts—earthy matter, represented by the phosphate of lime, etc., and the animal matter, represented by the cartilage or gristle. Children's bones are more easily bent than those of adults, because there is a larger quantity of the animal than of the earthy matter, but these two substances become more evenly balanced in adult life, though the balance is again lost in old age, when the animal part of the bone wastes away to some extent, and the earthy remain in excess, and thus the bones become very brittle, and old people break them very easily. Then the outside of a fresh bone, such as you can see any day in the butcher's shop. Why is it smooth ? You rub it, and it is like rubbing your finger up and down satin ; but if you rub one of those bones belonging to this skeleton, it is rough and harsh, and you naturally ask, are these two kinds of bone alike ? All bone in life, and before it has been carefully prepared for keeping, is smooth like satin, because it is covered with a very fine skin or membrane, which fits closely over it, and this membrane is most essential to its life ; it contains the bloodvessels, and therefore the food supply of the bone. If you deprive a bone of this membrane, it will die as surely as you would if your heart was taken away. Sometimes if the bone has been injured, as for instance, a severe kick on the shin-bone, this membrane dies, and that part of the bone which has lost its membrane dies, and will get quite loose from the rest of the shin-bone and come out, and in its place there will be new bone made ; for Nature will renew bone, under most circumstances, though, as you remember, she will not renew the cartilage at the ends of joints. The bones of a skeleton have merely no membrane—they are dead ; so would yours be had they none.

### Bunchy.

"Now, you'll promise, Bunchy, not to stir downstairs while I'm away ?" said careful John Price, the widower who had so lately lost his wife—the poor, ailing wife who had seemed so glad to slip away out of this toilsome, troublesome world, even though she had to say farewell to a tiny, peevish infant as well as to John and Bunchy.

"I promise, Dad," answered a sedate, small mortal with wide, earnest eyes, who, seated on a stool, was rocking the cradle gently, "I promise not to leave baby until you come home."

"That's my good Bunchy !" and John Price went off to his work contented, for his little woman had never been known to break her word.

She was a curious little soul, Bunchy. So short, so roundabout, and so slow in her movements, she was hardly like a child at all. Her small face was always puckered up with anxiety about some small point connected with the humble household, for had not Bunchy stepped into the mother's place, and who ever knew a mother with a smooth, placid face ? As yet, John Price had not missed his lost wife, in the way of his daily comforts, that was, for the little maid took careful heed to faithfully copy, as well as she was able, the old homely routine. Sometimes housekeeping was not so easy, though ; the baby brother was a handful in himself, always troublesome, always wailing, poor little scrap. But staunch Bunchy never complained nor murmured. At the worst, when all things would go wrong, and baby sternly refused to be comforted *anyhow*, she would whisper to herself mother's last intelligible words: "*You* will see after baby, won't you Bunchy?" and they always seemed just the help she needed.

"I do wish it was time for Dad to come home to supper," said the tiny woman, over and over again, as the day wore on to evening. Her little preparations were all made, but, still, no father came, and John had never been so late before. Bunchy was not old enough to feel that feverish anxiety which consumes, when the expected one lingers, until the whole heart becomes faint—the young are so trustful—so she sat down to her post beside the cradle to wait patiently; but waiting in the still quietness soon made the little one drowsy; the next thing to happen was a slipping and sliding nto the land of dreams, and the hours flew past in the hushed room.

A hissing patter, like hail, mingled with a strange, incessant, roaring sound; a jumbling confusion of hoarse cries; a bright red flare lighting up every corner of the little room, showing plainly Dad's untouched supper; the bullfinch, all in a flutter, in its cage; the sleeping baby in the cradle; Bunchy fell awake, suddenly, to wonder what all this meant. A thunderstorm, and how it rains! was her first thought. But, presently, she knew the patter came from no clouds, nor was the strange roaring, thunder. Loud shouts of "Fire! Fire!" from below told her what was the matter. Running to the window, Bunchy looked down upon a strange scene. A lurid light showed her busy steamers working their hardest; firemen rushing here and there, their helmets shining in the glare; water playing on the window, pattering on the roof; and a packed mass of white upturned faces.

"Come down, come down!" shouted one of the crowd, catching sight of the little head at the window.

"There's a child up there! Save her!" shouted another.

Go down! Leave the baby! Oh, no; Dad's last words were: "You'll promise, Bunchy?" So Bunchy, turning quietly from the window, sat down on her stool and hushed the restless infant. Higher, higher crept the flames, more densely rolled the smoke, making its way under the door, filling the little throats of Bunchy and her charge, so that they coughed incessantly.

"I heard somethin', Bill."

"So did I, mate; it was a cough." And two brave firemen plunged into the room.

"No, no, no!" shrieked Bunchy, wildly, as one of them swept her off her stool. "Dad said I wasn't to leave baby, and I mustn't."

"All right, the little 'un shall come too, never fear."

As the fireman spoke, a mighty crash sounded behind them. The staircase had fallen, and retreat was cut off. With a cruel roar the flames were in the very room, crawlng, then leaping upon them, greedy to embrace them.

"There they are at the window," shouted the crowd. 'Quick! The fire escape!"

Then came a pause of agonised suspense, a hush. Bravely, cautiously, the firemen did their work, while the crowd held its breath. They are saved at last, and a cheer that is deafening goes up! The babe is untouched, but the little lass is badly burnt. So is the fireman who carried her. "You see, she would have the little 'un saved first, and the flames got hold of the hindmost," the people tell one another, half sobbing, half cheering.

Dinner is over in the ward, and everyone is ready for the event of the day—the coming of the visiting physician. Punctually, the great man enters, followed by his class. From bed to bed they pass. Then, beside a cot there is a long halt, and the students crowd closely round. The patient—a tiny human cocoon, swathed in cotton-wool—looks wonderingly at the circle out of her bright eyes, and a wild desire to cheer her runs through the students as she does so, for it is Bunchy, and they all know her story. The little woman has been badly burned, but she is going to live, says the great man, and he silently thinks that the world would be the poorer if she did not. By-and-by the procession passes to other cases, but still the visiting physician's thoughts are full of the tiny heroine; and that evening, in the dusky quarter of an hour before dinner, he tells the tale to another little maid seated upon his knee. Between those two it is arranged that great things shall be done for Bunchy and her baby, when she can creep out of the cotton-wool back to life. A certain employment is known to be vacant far away in the beautiful, fresh country. A little cottage dressed in ivy goes with it. The only thing wanted is a steady, downright good man, and the physician thinks he has got that man.

And Dad, who kept his brave little daughter so long waiting at her post that death itself well-nigh relieved her? There had been an accident that afternoon, a fellow-workman had fallen off a scaffolding, and John Price had volunteered to take him home, then had remained to tend him. An every-day occurrence, but as the father gazes on Bunchy in the hospital cot, he feels that his Samaritan work has been done at a nearly terrible cost—nearly, not quite, thank God, for the little maid is to be spared to him.

### The Children's Hospital.

Written for the opening of the new Children's Hospital, Leicester.

Religion, fettered by our human creeds,
Is yet divine, and, when occasion needs,
Untrammelled Goodness crowns her gracious deeds.

As Constantine saw wonders in the sky,
And after listened to the children's cry,
Religion sees Love's ensign lifted high.

She hears her Lord's command as clear to-day
As when the Syrian mothers heard Him say
"Suffer the children," and she doth obey.

The Dante of "this desert life" hath sung
That though Love's power is told by every tongue,
The world is ofttimes cruel to the young.

'Twas in "Our Forest of the Past" he heard
The helpless children wail, yet no man stirred
To soothe with tender touch or gentle word.

The poet's darksome vision dies away,
And in the sunshine of this morn in May
Our children walk where kindness bears the sway.

How blest are they who, faithful to the Light,
Built this House Beautiful, where, day and night,
True hearts will try to save our buds from blight.

May richest blessings on the sufferers rest,
And when the greensward by their feet is pressed,
Let glowing Health attend each little guest.

To-day our joy must be to mark this throng
Of darlings, blithe as birds the flowers among,
And hear their laughter, sweet as sweetest song.

Alfred Charles Brant.

### Everybody's Opinion.

*Correspondence on all subjects is invited, but we cannot in any way be responsible for the opinions expressed by our correspondents. No communications can be entertained if the name and address of the correspondent is not given, or unless one side of the paper only be written on.*]

### SUPERANNUATED AT FORTY-THREE.

Nurse E. writes: As a reader of your valuable paper, may I ask you to insert this letter, which will I I hope draw the attention of medical men and others to a subject which has, I believe, already been a matter of grave complaint. I, an experienced nurse, holding good testimonials from the London hospitals, can find no employment and am only snubbed and rejected because I have unfortunately lived to the age of 43, which old age makes any application useless in these days. I am one of the first thousand who joined the list of the National Pension Fund, and I, with many more, I fear, who depend upon their labour to live and have no other resources, cannot, unless things alter, continue its membership, so my object is defeated. It is a stern question what we poor old nurses of 40 or 43 are to do. There are many in the ranks of despair like myself who try hard and tramp hard for work and get none. Harder still is it to see our zealous labour outdone, and our experience thrown to the wall, for our youthful sisters to take our place, because we are no longer young. The fact is, ladies take all vacant posts immediately, which ought to be filled by more mature women, who, from previous services, should have a claim. So we are weeded out, thus throwing a large number into the fields of unemployed labourers.

[Will Nurse E., and any nurse similarly situated, send a short statement to the Editor, giving her age, place where trained, number of years training, copy of certificate, and a short history of her employments with dates of engagements, and work since the completion of her training. A photograph should also be sent, if possible.]

### Hymn for a Hospital.

Angel of love, for every grief
Its soothing balm thy mercy brings,
For every pang its healing leaf,
For homeless want, thine outspread wings.

Who is our brother? He that lies
Left at the wayside, bruised and sore:
His need our open hand supplies,
His welcome waits him at our door.

Here stand the champions to defend
From every wound that flesh can feel;
Here science, patience, skill shall blend
To save, to calm, to help, to heal.

Father of Mercies! Weak and frail,
Thy guiding hand Thy children ask;
Let not the Great Physician fail
To aid us in our holy task.

Source of all truth, and love, and light,
That warm and cheer our earthly days,
Be ours to serve Thy will aright,
Be Thine the glory and the praise.

From "Before the Curfew," by O. W. Holmes.

Those who work faithfully will put themselves in possession of a glorious and enlarging happiness.—Ruskin.

### Vacancies.

The following vacancies are announced:

**Matron.**

St. George-in-the-East, £60.

**Sisters.**

Bolton Infirmary.
Manchester Royal Infirmary, £40.

**Nurses.**

Barony Parochial Hospital, Glasgow (two), £24.

Borough Fever Hospital, Sheffield (several), £25.

Bradford Fever Hospital (two), £23.

Bridgwater Infirmary, £22.

Chester Fever Hospital.

General Nursing Institute, Covent-garden (several).

Hampstead Home Hospital(several) £20.

Hanwell Ophthalmic School (five assistant), £18.

Hospital for Consumption, Brompton (several), £25.

Hospital for the Paralysed and Epileptic, Bloomsbury (two), £25.

North Devon Infirmary (several), £20.

Northern Fever Hospital (several), £30.

North-Western Fever Hospital (several), £22 and £30.

Norwood Cottage Hospital, £22.

Prestwich Nurses' Home (several).

Rochdale Infirmary, £22.

Southport Nurses' Home (several).

St. Mark's, City-road, £25.

Victoria Home, Bournemouth (several), £25.

Workhouse Infirmary Nursing Association, W.C. (several).

**Probationers.**

Bristol Hospital for Sick Children.

Hertford Infirmary, £12.

Northampton Nursing Institute.

Southport Infirmary.

St. Helens Fever Hospital.

### Notes and Queries.

To Correspondents.—1. Questions may be written on post-cards. 2. Advertisements in disguise are inadmissible. 3. In answering a query please quote the number. 4. If a private answer is desired, a stamped addressed envelope must be forwarded.

Notice.—We must really ask our correspondents to be more particular in enclosing name and address. Constantly we receive queries or letters with no name attached.

**Queries.**

(81) Is there a nursing institution or hospital in Chili where English nurses are employed? *Nurse M.*

(82) *X.* asks: Can anyone tell me where an old man, between 45 and 50 years of age, can be received where he will be looked after kindly. Is there any home for aged men? He is an invalid (but not helpless). He can only be kept in clothes and pocket-money.

(83) Can anyone recommend a good "case book," suitable for use in a small hospital, and tell me where it can be brought? *Nurse Jane.*

(84) *A. J. R.* asks: 1. Where one can obtain waterproof (usual holland coverings objected to) overalls for use of visitors to Infectious Diseases Hospital. 2. The best sources from which to obtain nurses for shorter or longer periods for Infectious Diseases Hospital within the suburban district?

(85) *S. T.* wishes to know if anyone can tell her of a second-hand bookstall where medical books suitable for nurses can be obtained, such as Dr. Domville's Manual for Hospital Nurses, Lucke's Lectures on Nursing, or Croft's Lectures?

*Daisy* asks: Can you give me any information respecting the Government hospitals abroad, especially in South Africa, and how nurses get appointed to them, and to whom do they apply?

### TO PRIVATE NURSES.

This knowledge of character is essential to success. Your opportunities for its study will be varied and ample, but the way lies through the thicket of human infirmity, the quicksands of passion, across the desert of blighted hopes and disappointed aspirations, to be traversed oftentimes in pain and weariness, with temper tried and patience worn to the limit of forbearance. It is a tree of knowledge whereof the fruit is bitter to the taste, but it has in it the issue of success, and success is power. When you make your entrance into that jealously guarded circle, the family, your first duty will be to study, quietly and closely as may be, the idiosyncrasies of the patient. Here you will not infrequently be hampered and embarrassed by well-meant, but often injudicious, interference and suggestions from relatives and friends. There are few families which do not number among its members someone who has the reputation of profound knowledge in the treatment of all diseases, and who are wont to be considered, and think themselves oracles, fully competent to advise the attending physician, and qualified by an abundant lack of previous experience to assume control of the invalid and the sick-room.—*Dr. C. E. Simmons.*

